# Spatio-temporal pattern evolution of green development efficiency in Northeastern China and its driving factors

**DOI:** 10.1016/j.heliyon.2024.e32119

**Published:** 2024-05-31

**Authors:** Pengting Duan, Peng Du, Ziyun Ruan

**Affiliations:** School of Geographical Sciences, Liaoning Normal University, Liaoning Province, Dalian, 116029, China

**Keywords:** Green development, Evolution, Driving factors, Spatial durbin model, Northeast China

## Abstract

Scientific analysis of green development efficiency is crucial for promoting healthy green development at home. The subjects of this study were 181 counties in three provinces in Northeast China. As a first step, the Super-SBM model is utilized to estimate the efficiency of 181 counties from 2006 to 2020; in addition, spatial autocorrelation analysis is applied to assess green development efficiency, spatially and temporally, of 181counties; and to examine the driving factors and spillover effects associated with the efficiency of 181 counties, the Spatial Durbin model (SDM) is utilized. The conclusions show that 181 counties have low levels of green development efficiency and are all on a downward trajectory. Liaoning Province has the highest level, Jilin Province has the second highest level, and Heilongjiang Province has the lowest level. According to the geographical distribution, the locations with high and very high green development efficiency are roughly located in Mohe City, Huma County, Xunke County, Daqing Municipal District, Harbin Municipal District, Changchun Municipal District, Wafangdian City, Dalian Municipal District, and Zhuanghe City. There is a favorable spatial connection of efficiency across regions, but the positive spatial agglomeration trend first diminishes and then gradually increases. Economic development, industrial structure, policy regulations, and environmental protection play significant roles in economic development, industrial structure, policy regulations, and environmental protection. The contribution of this essay is of paramount importance for understanding the status quo and potential for green development in different counties in Northeast China and for realizing coordinated regional green development.

## 1 Introduction

Northeast China has played a crucial role in China's economic and social growth for many years. In response to the national push for rapid industrial expansion, the region has heavily exploited non-renewable resources for over five decades, leading to a critical situation of resource depletion in the 1990s that continues to worsen. The strategy of revitalizing the aging industrial bases in Northeast China was first put forth by the CPC Central Committee in 2003, then it was promulgated in 2016, with “green” as one of its key development concepts. The 19th Party Congress report in 2017 clearly showed that “we need to realize the modernization of harmonious coexistence between humans and nature, and the 14th Five-Year Plan and the 2035 Vision stated that an ecological civilization system should be built. The Chinese economy has entered a new stage of development. In the new process, it is necessary to continually enhance the utilization efficiency of energy and resources, reduce waste emissions, promote economic development toward high-quality, intensive transformation, build a resource-saving and environmentally friendly society, and promote green development. Green development is an essential component for China to reduce carbon dioxide emissions and become carbon neutral; it is also a way to realize economic transformation and high-equality development. Green county development is crucial worldwide. Therefore, it is particularly urgent to calculate the efficiency of county green development in the three provinces of northeastern China and examine their geographical and temporal fluctuations and influencing factors.

Green development efficiency is a further in-depth study of the expansion of green development. Considering environmental costs and resource inputs, green development efficiency is the ratio of the relationship and intrinsic linkage between the output and input of a country or region in economic activities [1,2]. The study of green development efficiency deepens green development research. Scholars have devoted a great deal of attention to green development efficiency, which mainly focused on how to realize and develop a green economy based on current conditions in the early stages, and then gradually expanded to the study of green development efficiency, attempting to introduce various variables and models to measure and make predictions for the future from the perspective of inputs and outputs [3,4]. Studies on green development efficiency generally base their measurements on environmental and economic indicators [5,6]. Numerous academics have examined not only efficiency measurement but also the features of the temporal and spatial distribution of green development efficiency and the factors that influence it. The study of green development focuses on the following aspects.

With regard to regional selection, the expansion from national [7], provincial [8,9], and urban clusters [10] to prefecture-level cities [11] began, with increasing attention being paid to special regions, such as the old northeast industrial base [12], the Yangtze River Economic Belt [13], the Yellow River Basin [14], and cities with a strong resource base [15]. The indicator system has changed from a single type of indicator that emphasizes one-sided economic development to a composite indicator that comprehensively considers economic, social, and environmental factors [6], which has evolved the elaboration of green development efficiency [16], evolution characteristics and development prediction [17], measurement of total factor productivity, and corresponding index decomposition [18]. Recently, the input-output analysis method has been used to assess the construction of human, capital, and resource inputs, along with desirable outputs such as economic, social, and environmental benefits. It also accounts for undesirable outputs like environmental costs, providing distinct advantages in evaluating their overall impact. This has been studied by many scholars [19]. As for the mechanism of action, according to the “first law of geography,” Tobit models [7], spatial econometric models [14], geographically weighted regressions [15], and other methods are mostly utilized to analyze the impact of environmental conditioning, technological advancement, industrial adjustment, economic development, and other driving factors on green efficiency. Typical literature and their findings are shown in Table 1.

**Table 1:** Typical literature and findings.

Author	Regional Selection	Indicator system	Mechanism of action	Main findings
Tian et al.(2022) [7]	277 prefecture-level cities, China	Capital, labor, technology inputs; socio-economic gains; environmental pollution	Tobit regression model	Cities generally have a low degree of green development efficiency, albeit it has recently increased. Population size, economic development level, and the share of tertiary industry all significantly positively affect green development efficiency.
Yang(202) [9]	Shanxi Province, China	Labor, capital, resource inputs, green GDP benefits	Gray correlation model	Shanxi Province's green development efficiency has a cyclical upward tendency; the degree of pollution prevention and control has a higher impact on the efficiency of green development.
Ma(2022) [13]	Yangtze River Economic Belt, China	Labor, capital, resource inputs; economic returns; environmental pollution	Decision Testing and Evaluation Laboratory (DTEL) method	The Yangtze River Economic Belt's green development efficiency is generally increasing, exhibiting the downstream > upstream > middle reaches distribution characteristics; investments in scientific research, industrial structure, opening up to the outside world, environmental regulation, and vegetation coverage rate all significantly improve the green development efficiency.
Qian(2021) [14]	Yellow River basin, China	Labor, capital, land, water, energy inputs; economic, social, environmental benefits; solid, liquid, gas pollution	Spatial econometric model	Long-term improvements are typically observed in the green development efficiency of cities along the Yellow River Basin; the green development efficiency is positively impacted by the degree of economic development, technological innovation, and government backing.
Wang(2022) [15]	Resource-based city, China	Non-resource, resource-based inputs; economic, social, environmental benefits; environmental pollution	Geographically weighted regression models	Resource cities' green development efficiency demonstrates a positive development trend; the influence of green development is positively correlated with economic development, degree of external opening, urbanization, scientific and technological innovation capacity.

As a result of the literature review analysis, existing studies on measuring green development efficiency and the factors affecting it have the following characteristics. First, the research focuses on large regions of the country, special areas, urban agglomerations, and industries; however, few studies have focused on counties. The measurement of green development efficiency at the county level, the analysis of influencing factors, and the decomposition of spatial effects are a few studies that have been conducted. Research on green development efficiency still has scope for expansion. Second, in examining green development efficiency measurement indicators, scholars commonly prioritize labor, capital, and energy inputs in economic production. Regrettably, scientific and technological developments as inputs often receive insufficient attention. Additionally, ecological outputs, such as carbon dioxide absorption and emissions, are frequently overshadowed by economic output indicators. Third, when measuring the efficiency of green development, there was some overlap or crossover between the indicators and influencing factors. Some researchers use the same indicator as both an output indicator and influencing factor, which compromises the accuracy and scientific validity of their findings.

Generally, there is an opportunity for improvement, even though the findings of previous studies on green development efficiency are rich. First, most green development efficiency measures ignore “carbon emissions” as an undesirable output indicator that does not fully reflect the core meaning of green development. Relatively few studies have focused on spatial differences in green development efficiency and their reasons. Second, while the Tobit and standard panel regression models have been more frequently employed in studies analyzing the factors influencing green development efficiency, the spatial econometric model can compensate for the spatial factors that were overlooked in earlier studies by accounting for the interactions between various spaces or regions. Third, available research only examines the effectiveness of green development at the province level; research at the prefecture level, on the other hand, mostly focuses on specific city clusters or economic zones. Peace exists worldwide when nations are managed. To realize integrated regional and high-quality development, it is crucial to comprehend the existing status of green development and the potential for green growth in various countries. Research on the variations in the degree of green development efficiency in the counties of the three northeastern provinces remains scarce, although there are more studies on green development in Northeast China.

The marginal efforts made in this study are as follows: first, for fear of the inability of comparison when efficiency value is 1, we selected the Super-SBM model to develop an input-output index system that incorporates undesirable outputs and slack variables; and carbon dioxide absorption and emission are innovatively introduced, the index method, which can reflect economic and social growth in a more comprehensive way; second, we examined the spatial and temporal evolution features in 181 counties by taking advantage of spatial autocorrelation; finally, we utilized spatial Durbin model (SDM) to investigate the driving forces behind green development efficiency and its spatial spillover effects and propose logical countermeasures to help improve green development in 181 counties in the future.

The innovations in this study are reflected in the research objectives. The Yangtze River Economic Belt, Yangtze River Delta, Yellow River Basin, and other regions are the primary locations of empirical studies on China's green development efficiency that use urban agglomerations as their subjects. Presently, these studies focus primarily on provincial or urban scales in developed regions. To examine the degree of green development in the region, this study selected three northeastern provinces of the main national strategy as the study area, using 181 counties as a research unit. By selecting this range of options, previous scholars have examined the few and far-between options. Additionally, this study expands the research dimensions of the degree of efficiency of regional green development.

The second aspect is innovation in the selection of indicators. Gross domestic product (GDP) is typically used as a measure of the traditionally expected output indicators of the former, yet there are still certain limitations when it comes to studying the setting of this indicator alone. Consequently, this study draws on prior research findings regarding the consideration of environmental, scientific, technological, and social equity factors and introduces resource input indicators. The economic, social, and environmental gains introduced into the measurement of the indicators can make the final measured value of green development efficiency more persuasive and comprehensive, fully reflecting the development of goal-oriented green development efficiency. Most scholars use industrial wastewater, SO2, and smoke and dust emissions to measure undesirable outputs. Because the county needs to reduce net carbon emissions from both carbon emissions and carbon absorption in the context of carbon peaking and carbon neutrality, this study adds carbon dioxide absorption to the desirable output, and carbon dioxide emissions and PM2.5 average values are added to undesirable outputs.

The third aspect is innovation in the application of research methods. With reference to earlier research on efficiency value measurement, the angular and radial classic DEA model was employed as the primary technique. To assess the green development efficiency of the 181 counties in the three northeastern provinces, the Super-SBM model chosen for this study has the following main features: first, it accounts for unexpected outputs; second, it resolves the relaxation problem; and third, it does away with the Tobit panel regression model used by earlier researchers. Additionally, it builds a spatial econometric model based on the spatial dependence of the areas in the region to examine the factors influencing the green development efficiency of 181 counties in the three northeastern provinces. This research employs a combination of traditional and practical methodologies, resulting in more objective and policy-relevant empirical findings for the paper's research.

Constructing and improving the indicator evaluation system in line with the actual green development of counties in the three northeastern provinces is essential for analyzing contradictions in economic development, social progress, and ecological environment based on green development theory. This will help explore key breakthroughs in enhancing the quality and efficiency of green development and set the foundation for achieving the “dual-carbon” goal in counties. The disparities in development among counties reflect wider regional disparities in China. Measuring the degree of green development in counties is crucial for creating targeted regional green development plans. This highlights deficiencies in green development across different regions, streamlines development guidelines and optimizes the development process. Understanding the development patterns of local human-land relationships, identifying the strengths and weaknesses of each location, and gaining a comprehensive understanding of the local green development situation can be achieved through studying the spatial differences in green development efficiency and the influencing factors. A comprehensive analysis of green development effectiveness across counties in the three northeastern provinces will provide an objective assessment of the current state and shortcomings in green development. This assessment will help the government establish clear guidelines for the ecological development trend in the region and lay a scientific foundation for future ecological planning. Furthermore, proposing improvements to green development will aid in transitioning from rapid economic growth to high-quality development.

**Fig. 1:**
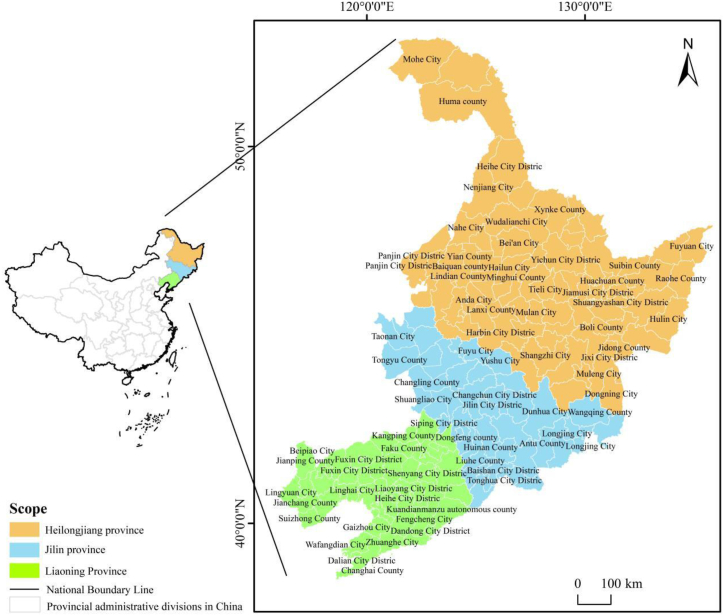
Overview of counties in three northeastern provinces of China.

The remainder of this article is organized as follows: Section 2 introduces the research approach, pertinent indicators, and data sources; Section 3 explores the spatial and temporal evolutionary patterns of green development efficiency in 181 counties; Section 4 analyzes factors that affect the efficiency of green development in 181 counties; and Section 5 contains the conclusion and discussion. The county profiles of the three northeastern provinces of China are shown in Fig. 1.

## 2 Methods, index selection, and data sources

### 2.1 Methods

#### 2.1. 1 Super-efficient SBM model with undesirable outputs (Super-SBM)

A method for making the optimal value judgment in economics is efficiency theory. In economics, efficiency is the amount of economic returns that different economic agents receive following the payment of a specific economic cost. For a given input, efficiency refers to the ability of resources to be fully utilized without being wasted. This implies that measurements of the link between inputs and outputs can be performed to optimize gains or reduce expenses. Given the traditional radial and angular nature of DEA methods, Tone [19,20] put forward a non-radial SBM model that improves the problem of equal scaling improvement and variable relaxation among variables. Tone then came up with the Super-SBM model, which permits the efficiency value of effective decision-making units (DMUS) to be ≥ 1. This helps to avoid the previous problem in which effective decision units could not be compared, and by assessing the DMUS more accurately and objectively, scholars have gradually recognized the benefits of the SBM model in assessing the efficiency of green development. Compared with the previous model, to avoid an objective comparison when the efficiency value is 1 by considering relevant relaxation variables and non-expected output variables, this study utilized the Super-SBM model to measure the green development efficiency level in 181 counties more accurately. The super-efficiency model in this study considers not only the various inputs used but also the desirable outputs, such as carbon dioxide uptake, and undesirable outputs, such as carbon dioxide emissions, combining multiple aspects (see Equation (1)).

(1) $$\rho =\min\frac{1+\frac{1}{m}\sum_{i=1}^{m}\frac{S_{i}^{-}}{x_{ik}}}{1-\frac{1}{q_{1}+q_{2}}\left(\sum_{r=1}^{q_{1}}\frac{S_{r}^{+}}{y_{rk}}+\sum_{t=1}^{q_{2}}\frac{S_{t}^{-}}{b_{tk}}\right)}$$

$$s.t.\left\{ \begin{array}{c} x_{ik}\geq \sum_{j=1,j\neq k}^{n}x_{ij}\lambda_{j}-S_{i}^{-}\\ y_{rk}\leq \sum_{j=1,j\neq k}^{n}y_{rj}\lambda_{j}+S_{r}^{+}\\ b_{tk}\geq \sum_{j=1,j\neq k}^{n}b_{tj}\lambda_{j}-S_{t}^{-}\\ 0<1-\frac{1}{q_{1}+q_{2}}\left(\sum_{r=1}^{q_{1}}\frac{S_{r}^{+}}{y_{rk}}+\sum_{t=1}^{q_{2}}\frac{S_{t}^{-}}{b_{tk}}\right)\\ \lambda_{j},S_{r}^{+},S_{i}^{-},S_{t}^{-}\geq 0\\ i=1,2,\ldots \;m;\\ r=1,2,\ldots q_{1};\\ t=1,2,\ldots q_{2} \end{array}\right.$$

In this case, ρ indicates the super-efficiency value, k represents the DMU measured; $\lambda_{j}$ is each DMU's weight; there are m input indications $x_{i}$ on each DMU; $q_{1}$ desirable outputs $y_{r}$; $q_{2}$ undesirable outputs $b_{t}$, m = 4, $q_{1\;}$ = 3, $q_{2\;}$ = 2. $S_{i}^{-}、S_{r}^{+}、S_{t}^{-}$ are slack variables for input $x_{i}$, desirable outputs $y_{r}$, and undesirable outputs $b_{t}$, respectively.

#### 2.1. 2 Spatial autocorrelation analysis

Counties are typically related to other counties or cities because they are interrelated and do not exist independently of one another. Traditional econometric models may incorporate certain missing variables, because they do not account for the interactive influencing linkages among counties. Spatial autocorrelation is an important geographical research method that uses a spatial autocorrelation index to reflect the potential degree of correlation and visualize the results. To examine the spatial agglomeration or differentiation traits of efficiency in 181 counties, this study applied global and local Moran indices. Among them, the global Moran's *I* shows overall similarity of efficiency values in spatial neighborhood areas, with values ranging from −1 to 1. When I>0 and is close to 1, there is a positive spatial autocorrelation and an obvious degree of agglomeration. However, when I<0 and close to −1, it manifests a negative spatial autocorrelation and the more obvious dispersion. The expression is (Equation (2)).

(2) $$I=\frac{n\sum_{i=1}^{n}\sum_{j=1}^{u}W_{ij}\left(X_{i}-\bar{X}\right)\left(X_{j}-\bar{X}\right)}{\sum_{i=1}^{n}\sum_{j=1}^{n}W_{ij}\sum_{i=1}^{n}{\left(X_{i}-\bar{X}\right)}^{2}}$$

I refers to the global Moran index, n is the total number of spatial units, or 181 counties; $\bar{X}$ stands for the mean value of n evaluation units in the 181 area; i is the green development efficiency value, $X_{i}$ is the i th county green development efficiency value, $X_{j}$ is the j th county green development efficiency value; $W_{ij}$ is a matrix of spatial weight, and this article uses the Queen adjacency matrix.

(3) $$W_{ij}=\left\{ \begin{array}{c} 1\\ 0 \end{array}\right.$$

$W_{ij}$ is a matrix of spatial weights. As spatial adjacencies, counties i and j are adjacent to each other when $W_{ij}$ is 1; when $W_{ij}$ is 0, counties i and j are not adjacent. The expression is (Equation (3)).

Additionally, local spatial autocorrelation can be applied to investigate the level of clustering of similar efficiency attribute values in counties and surrounding counties in the three northeastern provinces. If $I_{i}>0$ and reaches a significant level, this indicates that the assembly of high or low values of similar efficiency occurs around the counties. In contrast, $I_{i}<0$ exhibited a discontinuous distribution. $\bar{X}$ stands for the mean value of n evaluation units in the 181 areas, i is the green development efficiency value, $X_{i}$ is the i th county green development efficiency value, $X_{j}$ is the j th county green development efficiency value, $W_{ij}$ is a matrix of spatial weights, $s^{2}$ is the variance. The local spatial autocorrelation and variance expressions are as follows (Equation (4) and Equation (5)).

(4) $$I_{i}=\frac{\left(X_{i}-\bar{X}\right)\sum_{j=1}^{n}W_{ij}\left(X_{j}-\bar{X}\right)}{\frac{1}{n}\sum_{i=1}^{n}{\left(X_{i}-\bar{X}\right)}^{2}}$$

(5) $$s^{2}=\frac{1}{n}\sum_{i=1}^{n}{\left(X_{i}-\bar{X}\right)}^{2}$$

Further, this paper utilizes the $G_{i}^{*}$ index to identify cold hot spots in the green development efficiency of 181 counties, when $G_{i}^{*}$ >0, $G_{i}^{*}$ <0, $G_{i}^{*}$ = 0 and significant, it respectively denotes the presence of high value agglomeration, low value agglomeration, as well as random distributions in local counties. n is the total number of spatial units or 181 counties, $X_{j}$ is the value of the attribute of element j. The expression is Equation (6).

(6) $$G_{i}^{*}\left(d\right)=\frac{\sum_{j=1}^{n}W_{ij}\left(d\right)X_{j}}{\sum_{j=1}^{n}X_{j}}\left(i\neq j\right)$$

#### 2.1. 3 The spatial econometric model

Spatial econometrics examines how to conduct research on spatial interactions based on regression models of cross-sectional or panel data. According to previous analysis, an association exists between efficiency and spatial location in 181 counties. However, if a general panel model is adopted, spatial dependence between the study regions is ignored. Therefore, a spatial econometric model was employed in this study to probe not only the factors influencing efficiency, but also the dependence and heterogeneity of efficiency in 181 counties in three northeastern provinces. Spatial econometric models are usually divided into spatial error models, spatial lag models, and spatial Durbin models. The spatial lag model is a model in which changes in an area's explanatory variables are affected not only by changes in its own core explanatory variables, but also by changes in the explanatory variables of surrounding areas, and is most commonly used to examine spatial correlations, the expression is (Equation (7)); the spatial error model puts the spatial perturbations in neighboring regions into the regression term, reflecting the effect of spatial error perturbations in the surrounding regions on the explanatory variables in the region, which is primarily concerned with the spatial effect of the random disturbance term, the expression is (Equation (8)); by combining the spatial error model and the spatial lag model, the spatial Durbin model explains that a region's explanatory variables are not only influenced by its explanatory variables, but also by those of its neighboring regions, the explained variables, it accounts for the spatial impacts of random error shocks in addition to providing a good explanation of the spatial dependency of the dependent and independent variables, the expression is (Equation (9)). The spatial Durbin model was chosen to effectively capture the spatial spillover effect of green development efficiency.

Spatial lag models(SLM):

(7) $$y_{it}=\rho \sum_{j=1}^{n}W_{ij}y_{it}+\beta x_{it}+\mu_{i}+\lambda_{i}+\varepsilon_{it}$$

Spatial error models(SEM):

(8) $$\left\{ \begin{array}{c} y_{it}=\beta x_{it}+u_{it}+\mu_{i}+\lambda_{i}\\ u_{it}=\rho \sum_{j=1}^{n}W_{ij}u_{it}+\varepsilon_{it} \end{array}\right.$$

Spatial Durbin model(SDM):

(9) $$y_{it}=\rho \sum_{j=1}^{n}W_{ij}y_{it}+\beta x_{it}+\theta \sum_{j=1}^{n}W_{ij}+\mu_{i}+\lambda_{i}+\varepsilon_{it}$$

i and t denote county and year; $y_{it}$ signifies the explanatory variable for county i in year t ; ${\;x}_{it}$ denotes explanatory variables; $W_{ij}$ is spatial weight matrix; $\varepsilon_{it}$ and $u_{it}$ denote the random error terms; ${\;\mu }_{i}$ and $\lambda_{i}$ denote the spatial and temporal fixed effects respectively; ${lnX}_{1}$-${lnX}_{6}$ is the six explanatory variables after taking the logarithm; β is the constant term; $\beta_{i}$ is the estimated parameter for each variable; ρ is the spatial autoregressive coefficient, which mainly responds to the spatial lag effect; $\theta_{i}$ is the coefficient to be estimated; $W_{ij}y_{it}$ is the spatial lag term. The Lagrange multiplier (LM), likelihood ratio (LR), and Hausman tests were used to finalize the adoption of the dual fixed panel space Durbin model. In this study, the SDM is expressed as follows (Equation (10)).

(10) $$y_{it}=\beta +\rho \sum_{j=1}^{n}W_{ij}y_{it}+\beta_{1}{lnX}_{1}+\beta_{2}{lnX}_{2}+\beta_{3}X_{3}+\beta_{4}\;\ln\;X_{4}+\beta_{5}{lnX}_{5}+\beta_{6}X_{6}+\theta_{1}\sum_{j=1}^{n}W_{ij}\;\ln\;X_{1}+{\;\theta }_{2}\sum_{j=1}^{n}W_{ij}{lnX}_{2}+\theta_{3}\sum_{j=1}^{n}W_{ij}{lnX}_{3}+\theta_{4}\sum_{j=1}^{n}W_{ij}{lnX}_{4}+\theta_{5}\sum_{j=1}^{n}W_{ij}\;\ln\;X_{5}+\theta_{6}\sum_{j=1}^{n}W_{ij}X_{6}+\mu_{i}+\lambda_{i}+\varepsilon_{it}$$

### 2.2 Index selection

#### 2.2. 1 Green development efficiency index selection

**Fig. 2:**
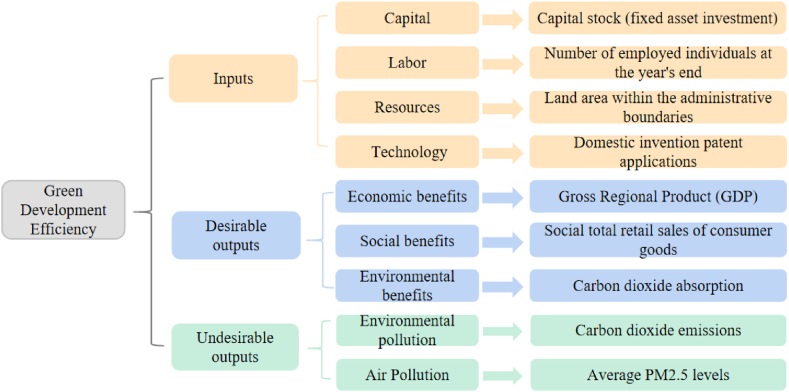
Input-output indicators for green development efficiency in three northeastern provinces.

This study considers existing research results, the concept of green development, and the current state of 181 counties. It integrates the National Development and Reform Commission of China Green Development Indicator System, Guiding Opinions on Building a Modern Environmental Governance System, and other documents issued by the General Office of the State Council, along with the “Nature-Economy-Society” (N-E-S) complex ecosystem theory. Following the principles of objectivity, comprehensiveness, operability, and data availability in index selection, an input-output index system on efficiency in 181 counties is constructed. The Super-SBM model consists of inputs, desirable outputs, and undesirable outputs. Inputs encompass capital, labor, resources, and technology. Capital is characterized by capital stock, specifically represented as fixed asset investment in each county. Labor input is calculated by the number of employed individuals at the year's end. Resources input is measured as the land area within the administrative boundaries due to lack of data on built-up areas. Science and technology advancement influencing green development is considered, along with the selection and approval of domestic invention patent applications. Desirable outputs include economic, social, and environmental benefits expressed as Gross Regional Product (GDP), social total retail sales of consumer goods, and carbon dioxide absorption, respectively. Undesirable outputs consist of environmental pollution and air pollution, indicated by carbon dioxide emissions and average PM2.5 levels in each county. A specific index system [[21], [22], [23], [24], [25], [26], [27], [28]] is illustrated in Fig. 2.

#### 2.2. 2 Variable selection for spatial economic models

**Fig. 3:**
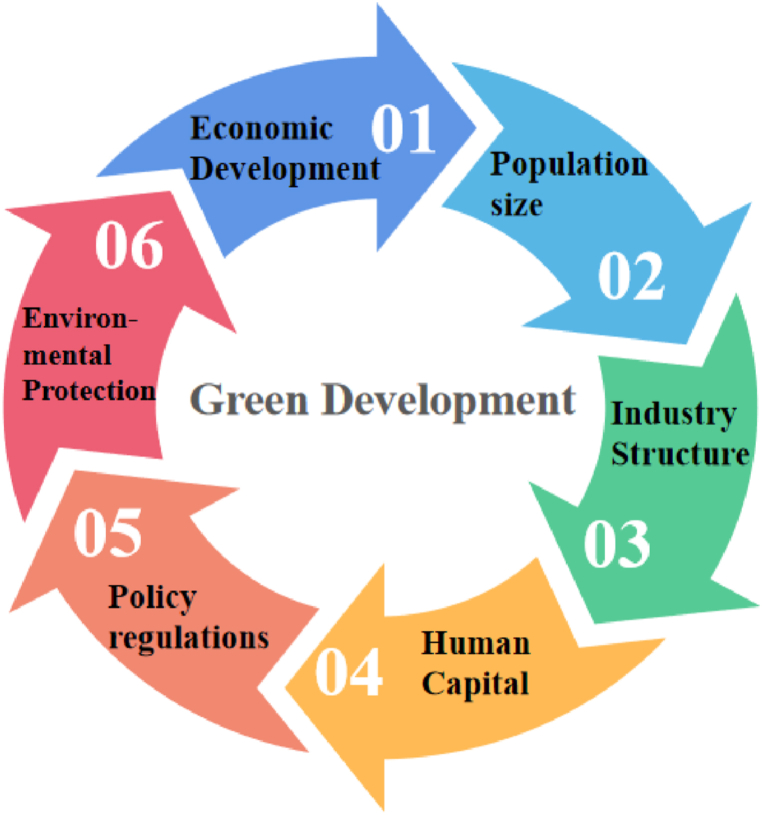
Factors influencing the efficiency of green development.

Considering the findings of recent studies and the current state of county development in the three northeastern provinces, this study selected driving factors from six elements: economic development, population size, industrial structure, human capital, policy regulations, and environmental protection. Economic progress level usually utilized Gross regional product per capita to represent [29,30]; because there are no data on the resident population, the level of population size is expressed in terms of household population data [31]; The industrial structure level is expressed by Secondary industry value added as a proportion of GDP [32]; number of students enrolled in primary and secondary schools serves as a measure of human capital [33]; the level of policy regulations is expressed in terms of local fiscal public budget expenditures [34]; based on the relevant references in this paper, carbon dioxide emissions' ratio for regional GDP is used to characterize carbon intensity to reflect the extent in which the regional emission reduction potential has been tapped [12], Table 2 and Fig. 3 provides an overview of the variables.

**Table 2:** Description of factors' affecting variables.

Explanatory variables	Definition	Units	Symbols
Economic Development Level	Per-capita GDP	Yuan	X1
Population Size Level	Household population	Ten thousand people	X2
Industry Structure Level	Secondary industry value added as a proportion of GDP	%	X3
Human Capital Level	Number of students enrolled in primary and secondary schools	Person	X4
Policy Regulations Level	Local fiscal public budget expenditures	Million yuan	X5
Environmental Protection Level	Carbon intensity	Million tons/billion yuan	X6

Regarding the selection of indicators, GDP is typically employed to show the degree of economic progress; however, owing to regional variations in population, GDP does not fully capture the picture. The link between per-capita income and environmental quality varies significantly depending on the stage of social development, as indicated by the environmental Kuznets curve. The impact of the economic development level on the effectiveness of green development and the two phases can be directly reflected in the per-capita GDP. Thus, per-capita GDP was used in this study because it is a better indicator of the degree of economic progress. Although an increased population results in a higher degree of economic development, it decreases per-capita resource ownership and exacerbates the conflict between the environment, resources, and population, making it challenging to increase the effectiveness of green development. Industry is the primary engine of the real economy and a significant component of secondary industry. To encourage industrial agglomeration and increase industrial scale, the optimization of industrial structure can efficiently distribute technology, capital, and other variables. It is used as a proxy measure of the industrial structure, in which the secondary industry occupies a dominant position in the three northeastern provinces of China. One of the key components of production is human capital, which affects the environment and causes positive externalities and factor effects that accelerate economic growth. The amount of human capital is expressed in terms of the number of students enrolled in primary and secondary schools, because talent mobility between cities will lead to the spread of ideas on a wider scale, which will have an impact on policymaking, production methods, and lifestyles in neighboring regions.

The direction and potential of green development in a region are determined by government policy orientation and green input. Green development is directly related to government investment levels. The efficiency of green development in cities is more immediately and significantly affected by government tax incentives and financial support programs, whereas firms are encouraged to minimize pollution emissions through government policy support and regulatory penalties. Nevertheless, it is important to remember that overzealous government macro-control interference in green development may result in government failure, which could have unfavorable consequences. Fiscal expenditure was used as a measure of government regulation to study the relationship between local fiscal expenditure and green development efficiency. The degree of environmental protection is a measure of the society's general well-being and the public's conscious understanding of environmental protection, in addition to the government's capacity to lead, organize, and control. Enterprises will allocate more funds to waste treatment and environmental protection in response to environmental regulations, which will successfully lower waste emissions and eventually increase green development efficiency.

### 2.3 Data sources

In this paper, 181 counties were the subject of research (including the municipal districts, counties and county-level cities after merging) in three northeastern provinces from 2006 to 2020, based on the base map in 2020, which was obtained from the Ministry of Natural Resources, PRC. The drawing review number is GS (2020) 4636, and without modification.

The fixed asset investment in the inputs is derived from the Heilongjiang Provincial Statistical Yearbook, the Jilin Provincial Statistical Yearbook, the Liaoning Provincial Statistical Yearbook, the China Urban Statistical Yearbook, and the Statistical Bulletin of the National Economic and Social Development of Selected Counties for the Period 2007–2021. Number of employed individuals at the year's end and Land area within the administrative boundaries were derived from the China County (City) Socio-Economic Statistical Yearbook, 2007–2021. Domestic invention patent applications were obtained from the State Intellectual Property Office of China (SIPO) (https://www.cnipa.gov.cn/). Gross Regional Product (GDP) and social total retail sales of consumer goods in the desirable outputs were derived from Heilongjiang Provincial Statistical Yearbook, Jilin Provincial Statistical Yearbook, and Liaoning Provincial Statistical Yearbook, 2007–2021. Carbon dioxide emissions and absorption data were derived from the China Carbon Accounting Database (https://www. ceads. net. cn/). The average PM2.5 levels from the Atmospheric Composition Analysis Group of Dalhousie University. Among them, the carbon dioxide absorption and emissions in 2018–2020 were made up by interpolation because of missing data, and the input-output indicators were measured and derived from raw data.

Among the influencing factors, the per-capita GDP, the number of students enrolled in primary and secondary schools, and the local fiscal public budget expenditures are derived from the 2007–2021 Heilongjiang Provincial Statistical Yearbook, Jilin Provincial Statistical Yearbook, Liaoning Provincial Statistical Yearbook, China Urban Statistical Yearbook, and the 2007–2021 Statistical Bulletin of National Economic and Social Development of Selected Counties; the household population is derived from China County (City) Social and Economic Statistics Yearbook 2007–2021; the secondary industry value added as a proportion of GDP is calculated from the secondary industry value added/total GDP; carbon intensity is calculated from carbon dioxide emissions/total GDP. To calculate the impact factors, the first five variables were logarithmised to eliminate the effects of heteroscedasticity.

## 3 Analysis of tempora-spatial evolution of green development efficiency

### 3.1 Evolutionary analysis of time series

The mean value of green development efficiency of the 181 counties in the three northeastern provinces from 2006 to 2020 ranged from 0.4401 to 0.6161, with a difference of 0.176 between the two; the standard deviation ranged from 0.3014 to 0.3961, with a difference of 0.0947 between the two; the minimum values range from to 0.0216–0.1277, with a difference of 0.1061; the maximum values range from to 1.2670–1.9686, with a difference of 0.7016. There was still a significant difference between the high and very high levels of efficiency, and there were more locations with low and very low levels of green development efficiency. Descriptive statistics of green development efficiency values are shown in Table 3. This suggests that the current model of economic growth in the counties of the three northeastern provinces is not well suited to address the issues of regional resource and energy scarcity, rudimentary production techniques, and economic inefficiency, and that a significant amount of improvement is required in the efficiency of regional green development.

**Table 3:** Descriptive statistics of green development efficiency values from 2006 to 2020.

Year	Mean	Std. Dev.	Min	Max
2006	0.5940	0.3168	0.0846	1.3137
2007	0.5923	0.3210	0.0599	1.3052
2008	0.5986	0.3014	0.1254	1.3285
2009	0.6161	0.3164	0.0941	1.3266
2010	0.6122	0.3193	0.1091	1.3035
2011	0.5637	0.3114	0.1053	1.2670
2012	0.4401	0.3073	0.0448	1.2805
2013	0.5925	0.3258	0.1277	1.3514
2014	0.6052	0.3230	0.0951	1.4074
2015	0.6115	0.3282	0.0769	1.4511
2016	0.5724	0.3439	0.0453	1.6151
2017	0.5687	0.3384	0.0508	1.5159
2018	0.6112	0.3534	0.0387	1.4414
2019	0.5574	0.3776	0.0342	1.4170
2020	0.5299	0.3961	0.0216	1.9686

**Fig. 4:**
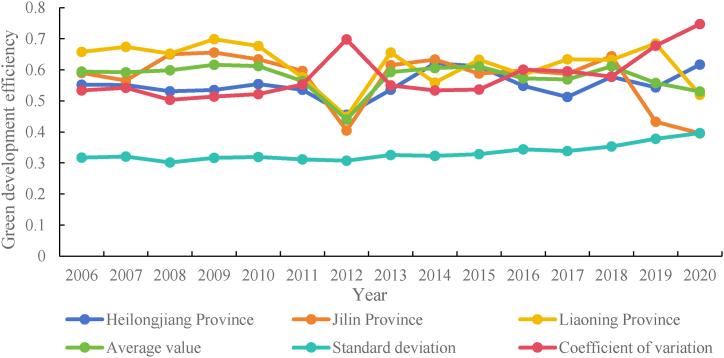
Mean, standard deviation, and coefficient of variation of counties in three Northeastern provinces of green development efficiency, 2006–2020.

The research used MaxDEA8.0 to determine the effectiveness of green development in 181 counties over a 15-year period. Moreover, the standard deviation and variable coefficient were combined to plot the mean change in efficiency in Heilongjiang, Jilin, and Liaoning provinces and the overall efficiency during 2006–2020. Where the standard deviation is utilized to characterize the absolute difference in efficiency in 181 counties and the variation coefficient illustrates the relative difference in efficiency among 181 counties. As shown in Fig. 4, the standard deviation and coefficient of variation curves indicate that the standard deviation remained stable during the study period but still showed an increasing trend, the coefficient of variation generally showing an increasing trend year by year, reaching a peak in 2012, and then decreasing and steadily increasing in 2013. This demonstrates that county-level disparities in the Northeast region's green development efficiency are growing both absolute and relative year over year.

Within the study period, Liaoning province had the highest efficiency value, followed by Jilin and Heilongjiang provinces. There was a fluctuating downward trend in efficiency in all three provinces, except Heilongjiang Province, which averaged less than 0.8, and both reached their lowest points in 2012. Since then, the 18th CPC National Congress has placed conservative culture in a prominent position, and sustainable development has become the strategic direction of regional development. During this period, the initial strategic adjustments resulted in a decline in efficiency. (Xu & Chen, 2022). In addition, since 2012, the three northeastern provinces have struggled with economic structural transformation dilemmas, economic decline in several cities and counties, slow fiscal revenues, low consumption levels by residents, and poor governance of energy conservation and emissions reduction, which have caused efficiency to decline. In Heilongjiang Province, the average efficiency fluctuated before 2012 and fluctuated up after 2012; the average value of green development efficiency in Jilin Province decreased overall in terms of volatility, whereas in Liaoning Province, the average green development efficiency fluctuated during the study period but remained higher than the average. The majority of Jilin and Liaoning Provinces are resource-dependent areas, where heavy chemical industries predominate. Owing to the long-term accumulation of an industrial structure and inadequate institutional mechanism reform, there are obstacles to regional green development. These three northeastern provinces have long been the main energy producers and consumers in China. Their economic growth is accompanied by the emission of various pollutants and their green development efficiency is naturally low. Thus, the existing green development efficiency of the three northeastern provinces has a larger size and greater flexibility of improvement.

### 3.2 Evolutionary analysis of spatial patterns

**Fig. 5:**
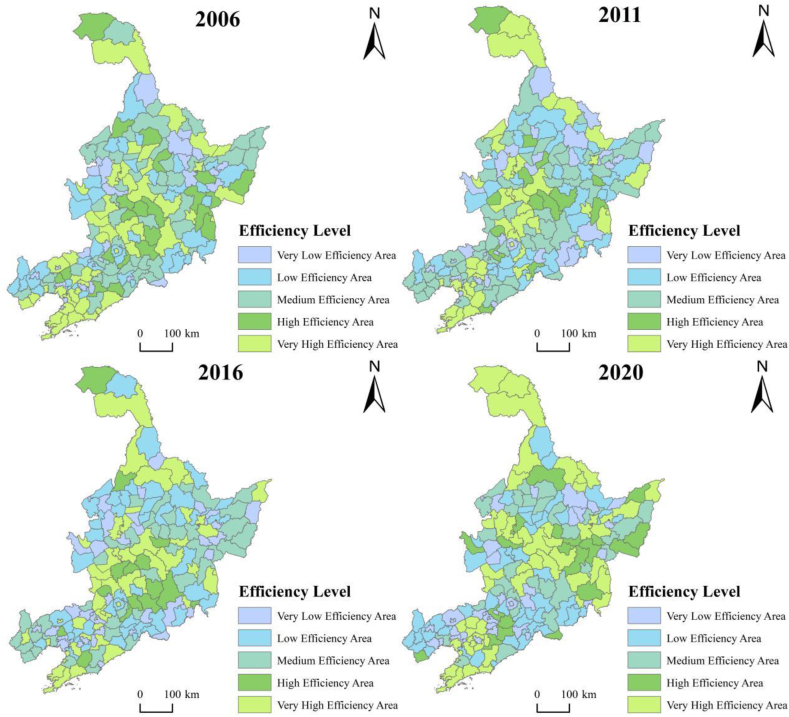
Spatial evolution of green development efficiency in three northeastern counties in 2006, 2011, 2016, and 2020.

In line with temporal evolution, our study also examined the spatial evolution features of efficiency. The primary objective was to choose a time period of every five years, and the efficiency data of 181 counties in 2006, 2011, 2016, and 2020 were selected. Using ArcGIS, the natural breakpoint means were utilized to classify the development efficiency in 181 counties of the three northeastern provinces and to create an average spatial evolution map of efficiency in the three northeastern provinces, as illustrated in Fig. 5.

The green development efficiency exhibitions of the 181 counties follow a band-like distribution. The efficiency of green development varied substantially among counties during the study period. There were more counties with high efficiency and very high efficiency in 2006; counties with medium efficiency significantly increased in 2011, and the medium efficiency area decreased in 2016; although certain counties' green development efficiency increased in 2020, the difference between the provinces' regions shrunk, and overall efficiency decreased. During the past four years, the low-and very low-efficiency areas were roughly located in Heihe Municipal District, Jixi Municipal District, Hegang Municipal District, Shuangyashan Municipal District, Qitaihe Municipal District, Da'an City, Tongyu County, Liaoyang Municipal District, Antu County, Helong County, Longjing City, and Chaoyang Municipal District. As these counties have different industrial structures and resource abundances, they face different development paths in terms of economic transformation and industrial structure adjustment. While the overall economic trend of the three northeastern provinces is sluggish, there is a lack of financial and technical support for transformation and development, uneven distribution of resources to support green development, and green development is limited [35,36]. However, since the revitalization strategy of the northeast was proposed, the four coal cities of Jixi, Hegang, Shuangyashan, and Qitaihe have remained true to their traditional and developing industries while also optimizing and adjusting their industrial structures. For instance, Hegang City depends on the graphite sector as a driving force for modernization and transformation to lessen the negative effects of the coal sector on the local economy. Overall, the results have been unsatisfactory. The percentage of energy-intensive and pollution-producing industries remains significantly higher than in other regions, and inadequacies in resource management and environmental governance further impede efforts to raise the bar for green development.

Highly efficient areas are located in Mohe City, Huma County, Xunke County, Daqing Municipal District, Harbin Municipal District, Changchun Municipal District, Wafangdian City, Dalian Municipal District, and Zhuanghe City. Within the study area, the surrounding counties are highly efficient, and it can be inferred that these counties have a significant driving and spillover effect on the surrounding counties. The Liao-Zhong-South City Cluster and Ha-Chang City Cluster were developed in the Dalian City District, Changchun City District, and Harbin City District. These districts have significant political and economic standing in Northeast China's former industrial base, are home to many high-quality production factors, and possess a distinct advantage in green development. Its industrial structure is becoming increasingly rational, forming clusters of industries, such as agriculture, biomedicine, equipment manufacturing, and automobile manufacturing, which are drivers of economic growth. Simultaneously, the tertiary sector is growing significantly and accounts for a far larger percentage of a city's total economy than other cities and municipalities. Changchun Municipal District has adopted a creative strategy for investment promotion by introducing high-tech businesses and stepping up efforts to foster interregional collaboration. Among them, some counties near the large and small Xinganling Mountains, such as Mohe City and Huma County, have higher green development efficiency because of their unique geographical location and climatic conditions. While their economic development level is relatively low, they have rich arable land sources, forest sources, wildlife, and strong technical support. Moreover, a higher level of agricultural mechanization and scale makes green development more efficient than in the rest of the province [37,38].

### 3.3 Spatial evolution based on correlation analysis

The global Moran's index is frequently utilized to examine the spatial connection between these explanatory variables, Moran's *I* scatter plot proposed in this research to examine the spatial correlation traits of each county's efficiency between 2006 and 2020, the results are shown in Table 4. Except for a few years, a fluctuating upward trend was observed for the global Moran's *I* after passing the significance test. The results showed an obvious positive spatial correlation and a trend of agglomeration distribution. In spite of the index values of individual years within the study period, it is found that the clustering development is generally characterized by a change in the shape of an “N.” Overall, Moran's of efficiency for the 181 counties exhibited an increasing, declining, and then increasing trend over the past 15 years. Additionally, this suggests that the positive spatial agglomeration trend is unstable, initially weakening and then progressively strengthening.

**Table 4:** The global Moran's I and test values from 2006 to 2020.

Year	Moran's *I*	*Z*值	*P*值
2006	0.078	1.733	0.083
2007	0.171	3.679	0.000
2008	0.120	2.626	0.009
2009	0.148	3.193	0.001
2010	0.159	3.436	0.001
2011	0.034	0.820	0.412
2012	0.006	0.240	0.811
2013	0.206	4.406	0.000
2014	0.112	2.444	0.015
2015	0.116	2.532	0.011
2016	0.062	1.417	0.156
2017	0.045	1.048	0.295
2018	0.114	2.498	0.012
2019	0.121	2.637	0.008
2020	0.267	5.693	0.000

**Fig. 6:**
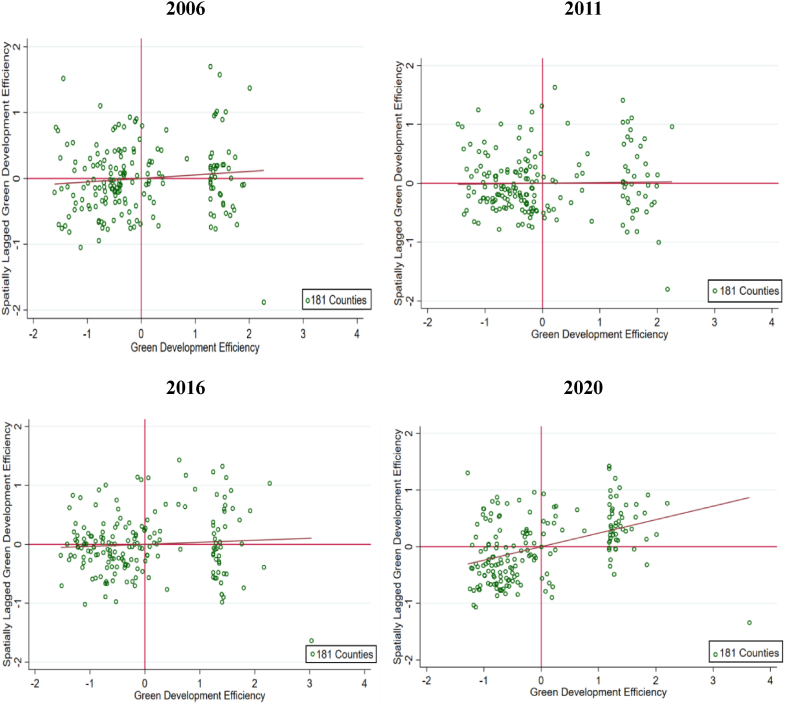
Local Moran scatter chart of green development efficiency at the county level in the three Northeast provinces in 2006, 2011, 2016 and 2020.

To estimate the spatial agglomeration of local efficiency, Moran scatter plots were selected for analysis for 2006, 2011, 2016, and 2020. Among them, high-high areas denote that the efficiency of a county and its surrounding counties are both high; a low-high area means that a county's efficiency is low and that of the next county is high; and low-low areas denote a county and its neighboring counties' ineffectiveness in promoting green development. The high-high and low-low regions indicate positive local spatial relationships at the local level, while high-low areas show a negative local spatial correlation. This can be seen in conjunction with Fig. 6. From 2006 to 2020, the number of counties in the first quadrant increased significantly, the number of counties in the second quadrant decreased, the magnitude of counties in the third quadrant remained largely unchanged, and the number of counties in the last quadrant decreased. There is no denying that green development tends to be more centralized in its dispersion, which also implies that despite the fact that the level of efficiency of the 181 counties has slowly approached a trend of balanced development, there is still an imbalance. Moreover, it can be observed that there is a progressive increase in the number of points in the first quadrant, which denotes an intensifying clustering of high values in the research units.

Using Moran's for global evaluation may have an adverse effect on local stability, for the purpose of further studying the spatial evolution of efficiency of 181 counties and which counties within the scope have a greater impact on the global spatial autocorrelation, the spatial pattern of efficiency of 181 counties are generated by calculating the local spatial correlation $G_{i}^{*}$ index of each county and classifying the local $G_{i}^{*}$ statistics in major years with the support of *ArcGIS*.

**Fig. 7:**
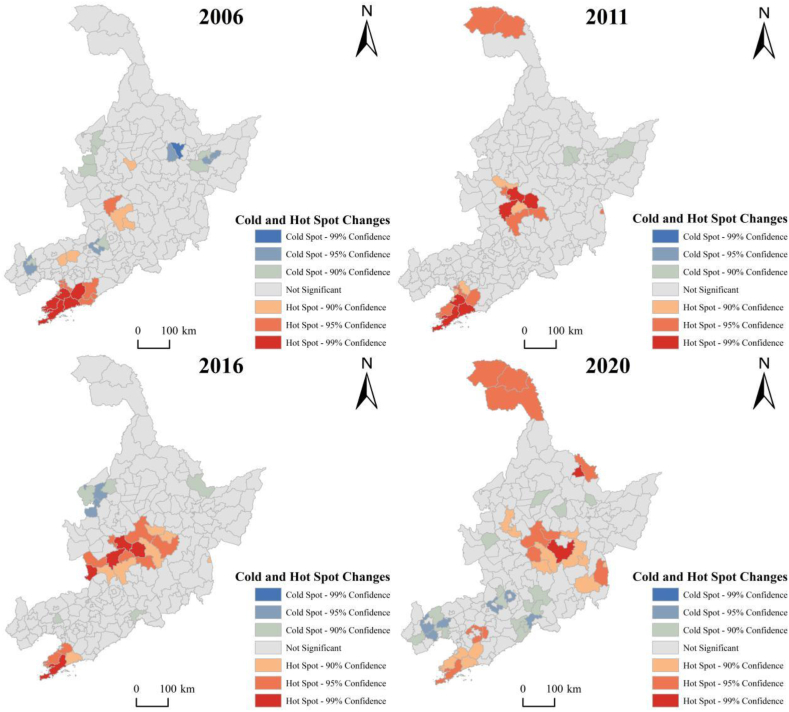
Cold and hot spot changes in green development efficiency in three Northeastern counties in 2006, 2011, 2016 and 2020.

Fig. 7 indicates that there are many counties in Northeast China where efficiency is not significant and there is a relatively stable distribution range. Liaoning Province's southeastern region and Jilin Province's central region, including the Songneng Plain and the Liaodong Peninsula, are considered hot spots for county efficiency. The hot pot areas over the past four years were mainly located in Nong'an County, Fuyu City, Yushu City, Wafangdian City, Dalian Municipal District, Gaizhou City and Zhuanghe City. Changchun Municipal District and Harbin Municipal District have a strong driving force and unique advantage in the process of green transformation. As centers of political, economic, cultural, social, scientific, and technological innovation as well as the development core of the Ha-Chang urban agglomeration, they have enormous potential for regional green development. Due to its abundance of tourism resources and high efficiency in using these resources, Dalian Municipal District has developed air, rail, and port transportation facilities. It has also constructed a nationwide transportation network that connects to the US, Australia, and Asia, and radiates to Russia, Japan, and Korea. This allows the district to quickly realize economic interaction with both domestic and foreign economies and encourages the redevelopment of tourism resources to realize green development.

The cold spot area of the county's green development efficiency has undergone significant adjustments. In 2006, it was situated in the following places: Qiqihar Municipal District, Tailai County, Zhenlai County, Daqingshan County, Nancha County, Huanan County, Jixian County, Shuangyashan Municipal District, Youyi County, Chaoyang County, Xifeng County; In 2011, it was situated in Daqingshan County, Nancha County, Youyi County and Fujin City; In 2016, it was situated in Longjiang County, Qiqihar Municipal District, Fuyu County, Tailai County, Luobei County, Beizhen City you, Baishan City District; In 2020, it was situated in the following counties: Hailun City, Tieli City, Hegang Municipal District, Da'an City, Jianchang County, Huludao Municipal District, Chaoyang County, Beipiao City, Yixian County, Xifeng County, Kaiyuan City, Huadian City, Huinan County, Jingyu County, Ji'an City. Generally, the cold spots of county green development efficiency fluctuated with time, but the overall trend remained stable.

## 4 Analysis of driving factors of green development efficiency

This study employs the LM, LR, and Hausman tests to select a spatial econometric model to further investigate the factors affecting efficiency in 181 counties, the results are shown in Table 5. First, both the Robust LM (lag) and Robust LM (error) tests reject the null hypothesis, demonstrating the need for geographical variables. The LR test also rejects the original hypothesis, indicating that fixed effects should be used, and the Hausman test also disproves the null hypothesis. We chose the spatial Durbin model, which contains individual and time fixations in both directions.

**Table 5:** LM, LR and Hausman test.

Test	W1
LM (lag) test	6.560**
Robust LM (lag) test	12.148***
LM (error) test	65.513***
Robust LM (error) test	71.101***
Spatial LR	74.51*
Time LR	1732.72*
Hausman test	106.26***

Note: *, **, and *** denote significance at the 10 %, 5 %, and 1 % levels, respectively.

### 4.1 Reference regression

Changes in the independent variables in the Durbin spatial model affect the efficiency of the local area and surrounding counties. In this study, direct, indirect, and total effects were categorized. Direct effects refer to the effect of these influencing factors on counties' efficiency, and indirect effects are spillover effects, which are the impacts of influencing factors on neighboring counties. In this calculation, we sum the direct and indirect effects to reflect the influence of numerous factors on efficiency across all counties. The specific effects are summarized in Table 6.

**Table 6:** Spatial Durbin model and effect decomposition results.

Variables	SDM Model	Wx	Direct effects	Indirect effects	Total effects
X1	0.11616*** (5.87)	0.00833 (0.27)	0.11701*** (6.04)	0.02936 (0.81)	0.14637*** (4.63)
X2	0.00787 (0.25)	−0.04897 (−0.90)	0.00298 (0.11)	−0.06425 (−1.16)	−0.06127 (−1.05)
X3	0.00007*** (3.53)	−0.00007* (−1.81)	0.00007*** (3.27)	−0.00007* (−1.69)	−0.00001 (−0.01)
X4	0.03340 (1.50)	−0.03097 (−0.68)	0.03531 (1.50)	−0.02526 (−0.43)	0.01006 (0.15)
X5	−0.06282*** (−3.54)	−0.04487 (−1.39)	−0.06770*** (−3.28)	−0.05848 (−1.54)	−0.12618*** (−3.07)
X6	0.00083*** (3.78)	0.00010 (0.17)	0.00084*** (3.96)	0.00024 (0.32)	0.00108 (1.31)
Rho	0.14200*** (4.93)				
R^2^	0.0596				
N	2715				

Note: Corresponding t-values are in parentheses; *, **, and *** denote significance at the 10 %, 5 %, and 1 % levels, respectively.

First, there are direct effects. The direct effects of the model on economic development, industrial structure, policy regulations, and environmental protection all pass the 1 % significance test with values of 0.11701, 0.00007, −0.06770, and 0.00084, respectively, meaning that at a confidence level of 1 %, when the control variables are increased by 1 %, they directly promote regional efficiency changes of 0.11701 %, 0.00007 %,-0.06770 %, and 0.00084 %, respectively. It is clear that economic development plays an active role; that is, the effectiveness of county green development projects can be considerably boosted by an increase in economic development. As the economy gradually develops to a certain level, the overall civilization of the city improves and the consciousness of environmental protection increases. In addition, the governance structure is strengthened, new technological means are invested to further enhance the effects on the environment, and economic development at this stage positively influences environmental development [39,40]. Generally, efficiency increases with reductions in policy adjustments, in accordance with the conclusions of Qian [13] and Qin and Liu [41]. Despite the fact that fiscal expenditure is one of the most important sources of funds for urban green transformation, it is severely constrained by a number of factors, such as the government budget, the use of investments, and the assessment of their effects. In the fight for regional economic development, local governments may prefer to invest funds in profitable regions that can create short-term economic growth, and the structure of local fiscal expenditure is irrational, while investment in environmental control efforts and green development is insufficient. As a result, the efficiency decreased. Moreover, in light of the notion of regional economic growth, the government should serve as a guide for the distribution of resources among regions; excessive intervention in resource allocation will have the opposite effect [42].

Second, spillover effects exist. The spatial spillover effect of green development efficiency is 0.14200, which is obtained through a significance test of 1 %, showing a large positive spillover effect; local effectiveness raises the effectiveness of the neighborhood. The industrial structure level has a spatial spillover coefficient of −0.00007, which passes the 10 % test, showing that the industrial structure level of this region hinders the improvement of the industrial structure level of adjacent areas. The levels of population size, human capital, and policy regulations also have negative spatial spillover impacts, indicating that improvements in population size, human capital, and policy regulations in a county inhibit improvements in efficiency in neighboring counties.

Third, indirect effects were observed. Based on the industrial structure, a strong spatial correlation impact only occurred between counties in the three northeastern provinces, as indicated by the indirect effect of the industrial structure level passing the 10 % significance test. According to the data in the table, at an acceptable confidence level, efficiency changes by −0.00007 % for every 1 % increase in the industrial structure level coefficient of the surrounding counties. The coefficients of population size, human capital level, and policy regulations level are also negative, consistent with the results of the spatial spillover effect, again suggesting that an increase in the level of human capital and policy regulations in a county will inhibit the improvement in the efficiency of green development in neighboring counties.

### 4.2 Robustness tests

Using various spatial weight matrix tests can strengthen the persuasiveness of the research findings to verify the validity of the SDM predictions of the variables that affect efficiency. Given that the geographic distance weighting matrix only considers the influence of geographic location and that some degree of regional barrier breaking has been made possible by infrastructural improvements, the spatial correlation in the context of economic distance is also particularly significant. The inverse distance matrix and economic distance matrix are used in this research instead of the roook adjacency matrix. Table 7 presents the results of the robustness tests. In general, the coefficients of the variables with significant positive and negative signs agree with the above, it can also be said that the empirical findings in this work are more reliable.

**Table 7:** Robustness test results.

Variables	(1) Inverse distance matrix	(2) Economic weighting matrix
X1	0.09719*** (4.98)	0.13236*** (6.87)
X2	0.00425 (0.13)	0.00300 (0.09)
X3	0.00007*** (3.88)	0.00007*** (3.45)
X4	0.03307 (1.49)	0.02319 (1.04)
X5	−0.07163*** (−4.02)	−0.06877*** (−3.92)
X6	0.00084*** (3.77)	0.00085** (3.85)
Rho	0.25841*** (2.62)	0.08568** (2.58)
R^2^	0.0172	0.1322
N	2715	2715

Note: Corresponding t-values are in parentheses; *, **, and *** denote significance at the 10 %, 5 %, and 1 % levels, respectively.

## 5 Conclusions and discussion

### 5.1 Conclusions

The essay selects 181 counties' data in three northeastern provinces from 2006 to 2020, revealing evolutionary patterns on a space and time scale and driving factors about green development efficiency of 181 counties, considering its spatial spillover effect, then drawing some conclusions: (1) Over the period 2006–2020, the average value of efficiency of 181 counties fluctuated. Liaoning Province had the best efficiency for green development, followed by Jilin and Heilongjiang Provinces has the lowest level. Each of the three provinces had an average efficiency value of less than 0.8, and all were experiencing a decline in efficiency. In terms of spatial distribution characteristics, the locations with high and very high green development efficiencies are roughly located in Mohe City, Huma County, Xunke County, Daqing Municipal District, Harbin Municipal District, Changchun Municipal District, Wafangdian Municipal District, Dalian Municipal District, and Zhuanghe City. Although some counties' green development efficiency increased and the difference between the provinces' regions shrunk within the research phase, efficiency generally decreased. (2) There is a favorable spatial correlation between efficiency and clustering distribution trends among regions. In general, its agglomeration development is characterized by an “N" shape change, The Moran's I of efficiency shows that over the past 15 years, counties in the three northeastern provinces exhibited a increasing, declining, and then increasing tendency in the past 15 years, indicating that this positive spatial agglomeration is not stable but instead weakens initially before progressively strengthening. (3) Several factors influenced the green development efficiency in 181 counties, including economic development, industrial structure, policy regulations, and environmental protection. Policy regulations was among the elements that negatively influenced efficiency in 181 counties.

This study proposes relevant policy recommendations in light of the above research: (1) Solidify the foundation of economic development and promote economic growth through green development. Each county's economic development status and modernization requirements must be clarified, and the corresponding policy framework must be improved. Encourage the transformation of industry and agriculture and strengthen the power of economic development through various means, such as governmental policy guidance and technical support from scientific research institutes. (2) Adhere to the connotations of sustainable development, adjust the industrial structure, and change towards a low-carbon, green, and efficient development model. The three provinces in northeast China should pursue green development as large industrial regions and as the first economic development regions in China. Green development is the way out to help enterprises transform to the “three low” model, reduce the emission of pollutants, eliminate backward industries promptly, and vigorously develop low-pollution industries, to accelerate green and sustainable economic development. (3) Improve environmental protection and pollution control capabilities and reduce carbon emissions. Improve the system of environmental governance by involving the government, enterprises, and the public, and improving the accountability system. Efforts should be made by the government, society, businesses, and public to enhance economic development in counties. Developing a circular economy, investing more in green technology, introducing environment-friendly technologies, and improving green technology levels By increasing funding for the R&D of new products, encouraging enterprises to develop and introduce green technologies, and increasing investments and subsidies for green science and technology innovation platforms, enterprises can be guided to establish the concept of green development. (4) Actively integrate the three northeastern provinces and help them become new banners of green development. Actively breaking down regional administrative barriers and promoting linked urban development. Considering that China will achieve a carbon peak in 2030 and carbon neutrality in 2060, each country cannot formulate green development policies independently. Counties with a high level of efficiency should achieve independent innovation and develop the three low-green industries based on accomplishing regional development goals. Counties with a medium level of efficiency should actively participate in high technology to accelerate efficiency by optimizing the industrial structure. Low-efficiency counties should consider whether there are omissions in county development planning and unreasonable industrial structures, and it is requested that counties with high efficiency distribute advanced production technologies and enterprises to counties with low efficiency to establish a good radiation linkage and mutual support situation, so that efficiency can be improved jointly.

### 5.2 Discussion

While the broader analysis of green development efficiency from various viewpoints produced by the existing literature provides a solid scientific foundation, it also has the following drawbacks. First, the county is a crucial location for regional administration, a significant area for the uploading and distribution of policies, a crucial area for high-quality development, and a fundamental building block for the design of primary functional areas. The majority of the literature currently in publication concentrates on the east-central region of China, important national strategic development regions, or the national level. Counties receive relatively little attention, with the three northeastern provinces receiving even less research on their counties and even less in-depth empirical analyses of their levels of green development. Second, the research is inconsistent and the sample period of the literature that is now in existence is somewhat short-mostly around 10 years. Third, the efficiency of green development is mostly measured by standard DEA or SBM models in the literature now in publication. This raises the issue because several decision-making units have simultaneous efficiency values of 1, making it impossible to compare and rank them further. Despite reading many academic papers and literature from both domestic and foreign authors, few studies have been conducted on the effectiveness of green development across many counties in the three northeastern provinces, and most studies to date have been conducted at the provincial level. The application of scientific measurements of green development efficiency to the analysis of the green development mode of the three northeastern provinces plays a crucial role in the green transformation of their economic development as it is an essential component of the process of building socialist modernization. The research period of this study was relatively long (15 years). The Super-SBM model was used to establish input-output indicators based on slack variables and undesirable outputs to avoid the impossibility of comparison when the efficiency was 1.

The county-level administrative unit is the most fundamental administrative unit in China's economic and social development at the county level, serving as a zone of integration between the country's agricultural and industrial sectors, as well as between urban and rural areas. As such, it can offer significant financial, land, labor, and other resources to support the growth of regional central cities and is essential to China's coordinated urban-rural integrated development, as well as the consolidation and protection of the people's fundamental power. Xi Jinping, General Secretary of the Communist Party of China (CPC), chaired a symposium on September 7, 2023, to promote the Northeast's comprehensive revitalization in the new era. He stated that the development of the ecological environment must be prioritized, environmental quality and ecological value must be continuously improved, and high-quality development must be supported by high-quality green environments. The Central Economic Work Conference, held on December 11–12, 2023, placed a strong emphasis on the need to fully embrace the work of the rk of the “three rural areas” and actively advance the establishment of an ecological civilization and low-carbon green growth. The Central Rural Work Conference, held on December 19–20, 2023, made several recommendations, including promoting the integrated development of urban and rural areas within counties; strengthening the comprehensive carrying capacity and governance of counties; and adhering to the development of agriculture through industry, quality, and greening. Evaluating the effectiveness of green development in China's northeastern counties is crucial, both theoretically and practically, because this region is China's ancient industrial foundation and a major grain-producing area.

It should be noted that although this study examines the spatial and temporal evolution traits and contributing factors to green development efficiency in the counties of the three northeastern provinces, there are still some limitations. Considering that the study area is small and data acquisition is difficult, the selection of indicator will inevitably be imperfect. In the future, we will continue to conduct both theoretical and practical research on green development, focusing on its comprehensiveness, coordination, and sustainability of green development. Taking a geographic and economic perspective on green development, we will continue to work towards perfecting its definition, theoretical basis, and realization path, and provide methodological support and unique research perspectives for solving ecological problems.

## Funding statement

This research received funding from by the 10.13039/501100001809National Natural Science Foundation of China [Grant No. 41701123].

## Data availability statement

Data will be made available on request.

## CRediT authorship contribution statement

**Pengting Duan:** Writing – review & editing, Writing – original draft, Formal analysis, Data curation. **Peng Du:** Writing – review & editing, Writing – original draft, Formal analysis, Data curation. **Ziyun Ruan:** Writing – review & editing, Writing – original draft, Formal analysis, Data curation.

## Declaration of competing interest

The authors declare the following financial interests/personal relationships which may be considered as potential competing interests:Peng Du reports financial support was provided by 10.13039/501100001809National Natural Science Foundation of China. If there are other authors, they declare that they have no known competing financial interests or personal relationships that could have appeared to influence the work reported in this paper.
